# Voiding urodynamics parameters for women with and without symptomatic pelvic organ prolapse

**DOI:** 10.61622/rbgo/2025rbgo52

**Published:** 2025-07-15

**Authors:** José Tadeu Carvalho Martins, Gisele Vissoci Marquini, Laura Aparecida Xavier de Abreu, Zsuzsanna Ilona Katalin de Jármy Di Bella, Marair Gracio Ferreira Sartori

**Affiliations:** 1 Universidade Federal do Espírito Santo Vitória ES Brazil Universidade Federal do Espírito Santo, Vitória, ES, Brazil.; 2 Universidade Federal de São Paulo Escola Paulista de Medicina São Paulo SP Brazil Escola Paulista de Medicina, Universidade Federal de São Paulo, São Paulo, SP, Brazil.; 3 Universidade Federal de Uberlândia Uberlândia MG Brazil Universidade Federal de Uberlândia, Uberlândia, MG, Brazil.

**Keywords:** Urinary bladder neck obstruction, Urodynamics, Urinary bladder, Urinary tract infections, lower urinary tract symptoms, Pelvic organ prolapse, Pelvic Organ Prolapse Quantification (POP-Q), Female

## Abstract

**Objective:**

This study aims to characterize voiding urodynamics parameters suggestive of bladder outlet obstruction (BOO) diagnosis in women with and without symptomatic pelvic organ prolapse (POP).

**Methods:**

Cross-sectional research. Patients were selected and clinically evaluated with anamnesis, pelvic organ prolapse quantification system and standard Urodynamic Testing, performed according to International Continence Society and International Urogynecological Association guidelines (noninvasive uroflowmetry, followed by invasive cystometry and a Pressure/Flow Study). Included criteria: women aged 18 to 94 years with and without symptomatic POP and lower urinary tract symptoms . Exclusion criteria: patients who were not clinically feasible, undesirable, or impossible urodynamic test, or patients with urinary tract infection or neurological lower urinary tract dysfunction.

**Results:**

Voiding urodynamics parameters suggestive of BOO diagnosis found in women with POP were: maximum flow rate (Qmax) in the uroflowmetry ≤12mL/s; detrusor pressure at maximum flow (PdetQmax) >20cmH2O. When evaluating the differences between patients with and without POP, it was observed that those who presented some type of pPOP were mean age (y) older (67.6 × 58.9); had higher post-void residue volume (mL) (85.9 × 33.9); higher PdetQmax (cmH_2_O) values (41.3 × 28.5); lower Qmax (mL/s) values on uroflowmetry (8.5 × 20.4), lower maximum cystometric capacity (mL) (325.7 × 381.2), lower bladder compliance (mL/cmH2O) (39.8 × 46.1) and lower Qmax (mL/cmH2O) in the flow/pressure study (8.4 × 18.0) (*p*<0.001).

**Conclusion:**

The voiding urodynamics parameters listed in this study allows the evaluation of variables suggestive of BOO diagnosis in women with and without POP.

## Introduction

According to International Continence Society (ICS) and International Urogynecological Association (IUGA), pelvic organ prolapse (POP) is a common and increasingly prevalent disease in adult and elderly women. The reported prevalence of POP varies widely (1-65%) based on whether its presence is ascertained by symptoms (1-31%), pelvic examination (10-50%), or both (20-65%).^(1,2)^

POP can trigger symptoms like a sensation of a "bulge in the vagina;" Lower Urinary Tract Symptoms (LUTS) encompass storage, voiding and post-micturition symptoms; bowel symptoms and even discomfort during sexual intercourse.^(3,4)^ POP should be carefully evaluated, including the qualification of urinary symptoms into storage and bladder emptying issues such as Bladder Outlet Obstruction (BOO).^(5,6)^

The prevalence of BOO in women is unknown and most probably has been underestimated. Moreover, there are no standard definitions for the diagnosis of BOO in women.^(7-11)^ Several authors have attempted to standardize urodynamic test values to define BOO in women. In addition, there is considerable heterogeneity between voiding urodynamics parameters.^(12-16)^

In clinical practice, nomograms are used for diagnosing obstruction; however, these are commonly designed for men, such as those from the ICS, Abrams-Griffiths, Schaffer, and urethral resistance factor.^(13)^ Blaivas et al.^(14)^ described a female nomogram using maximum flow rate (Qmax) in uroflowmetry and detrusor pressure at maximum flow (PdetQmax) in the pressure/flow study (PFS) as parameters. Generally, BOO is defined as a persistent, low, maximum "free" flow rate of <12 mL/s in repeated non-invasive uroflow studies, combined with high detrusor pressure at a maximum flow (PdetQmax) >20 cmH2O during detrusor pressure/flow studies (PFS).^(15)^

Because of this heterogeneity in existing data, voiding urodynamics parameters in women with POP are considered unrepresentative and unreliable and do not get any relevance as having any diagnostic utility. Probably, observed variation in voiding urodynamics pressures in women with POP compared to women without POP is due to the nonuniformity in the parameter being assessed as well as due to inherent differences in voiding physiology. Thus, this study aimed to analyze voiding urodynamic parameters suggestive of BOO diagnosis in women with and without POP.

## Methods

This descriptive, cross-sectional, quantitative, and comparative study was conducted at the Federal University of Espírito Santo ES-Brazil and Federal University of São Paulo, Brazil.

The study included women aged 18 to 94 years with Lower Urinary Tract Symptoms (LUTS) who were referred to the urodynamics department between January 2017 and December 2019. Exclusion criteria: patients who were not clinically feasible, undesirable, or impossible urodynamic test, or patients with Urinary Tract Infection (UTI) or neurological lower urinary tract dysfunction.^(17)^

The patients were invited to participate in the study, with clarification of the project and application of the inclusion criteria. Interviews, physical examination for the classification of Pelvic Organ Prolapse Quantification (POP-Q) System, and the urodynamic testing were carried out by a single researcher.

To determine the sample size, a pilot study with 85 cases was initially conducted to estimate the standard deviation for the source population. Once a standard deviation of 27.8 was identified, the sample size was calculated, with urethral resistance as the outcome variable, a confidence level of 95%, and a margin of error of 4 cmH_2_O. Thus, a minimum sample size of 186 individuals was estimated, as shown in the calculation below:


$$\begin{array}{c} \text{N}=(\text{Z}/\text{E}{)}^{2}.(\text{S}{)}^{2}\\ \text{N}=(1.96/4{)}^{2}.(27.8{)}^{2}=186 \end{array}$$

Where: N represents the sample size; Z is 1.96 (the standard normal distribution value for a 95% confidence level); E is 4 (defined as a safe margin of error for the examination) = margin of error; and S is 27.8 = standard deviation.

Standard Urodynamic Testing (UT) was performed according to IUGA and ICS guidelines. It involves noninvasive uroflowmetry, followed by invasive cystometry and a Pressure/Flow Study^(11)^ Patients were instructed to drink 4 glasses of 200mL of water and wait for the feeling of urge to urinate before starting Uroflowmetry (UF). The Dynapack MPX 816 multichannel urodynamics device from the company Dynamed was used. Cystometry was performed without the POP reduction maneuver (symptomatic POP). The urodynamic parameters analyzed, their units and reference values are listed in chart 1.

**Chart 1:** Voiding urodynamics parameters, their units, and reference values

Variables	Acronym	Unit	Reference value
Maximum flow rate in uroflowmetry	Qmax in uroflowmetry	mL/s	> 12 mL/s
Post-void residual volume	-	mL or % of maximum cystometric capacity	< 100 mL or < 20%
Maximum cystometric capacity	-	mL	350 – 500 mL
Bladder compliance	-	mL	≥ 30 mL/cmH_2_O
Detrusor pressure at maximum flow	PdetQmax	cmH_2_O	< 20 cmH_2_O
Maximum flow in the flow/pressure study	Qmax in the flow/pressure study	mL/s	> 12 mL/s
Classification of post-void residual volume	-	mL or % of maximum cystometric capacity	
Normal			< 100 mL or < 20%
Increased			> 100 mL or > 20%
Detrusor overactivity	-	cmH_2_O	
Present			>16
Not presente			< 16
Present at the end of micturition			>16
Leakage due to detrusor overactivity	-	mL	
Absent			< 1
Present			> 1
Urethral resistance	-	cmH_2_O/(mL/s)^2^	
Normal			< 0.2
Increased			> 0.2
Bladder outlet obstruction index	-	-	
Non-obstructive			< 20
Intermediate			20 – 40
Obstructive			> 40
Bladder contractility index (BCI)	-	-	
Strong			> 150
Normal			100 – 150
Weak			< 100
Blaivas & Groutz nomogram for female bladder outlet obstruction	-	mL/s	
No obstruction			Uroflowmetry > 20
Mild obstruction			Uroflowmetry 10 – 20
Moderate/severe obstruction			Uroflowmetry < 20

The patients were instructed to report their initial desire to urinate, at which point a physical examination was carried out to assess the degree of POP, standardized by the POP-Q system classification, observing the points Ba, Bp, C and D^(14)^ Points Ba and Bp were considered to be the points of greatest prolapse, in the Valsalva maneuver, respectively, of the anterior and posterior vaginal walls. Point C corresponds to the point of greatest prolapse of the apical wall (cervix or vaginal vault in hysterectomy patients) and point D corresponds to the insertion of the uterosacral ligaments, all measured during effort.^(14)^

The patients were grouped according to the Ba and Bp points, as follows: up to -1 (Ba/Bp<0), descent to zero (Ba/Bp=0); and descent above zero (Ba/Bp>0), with "0" representing the hymenal ring. For points C and D, descent was considered to be less than zero (C/D<0) and above or equal to zero (C/D≥0).^(6,7)^ Thus, patients with all points (Ba, Bp, C and D) less than zero were considered as a group "without externalization of POP" (control group).

The normality of variables was assessed using the Komogorov-Smirnov test with Lilliefors significance correction. Measures of central tendency (mean and median) and dispersion (standard deviation and range) were used for continuous variables and absolute and percentage values for categorical variables.

Regarding association tests between the independent variables and the outcome, Pearson's Chi-square test was used for qualitative variables. Due to the abnormality of the variables, the association of a quantitative and a qualitative variable was evaluated using the Mann-Whitney test. When the qualitative variable had three or more categories, the Kruskal-Wallis test was performed.

For all analyses, the significance level adopted was α < 5% and these were carried out using the statistical program IBM SPSS Statistics for Windows, version 22.0 (Armonk, NY: IBM Corp).

The study was approved by the Research Ethics Committee under number 1.842.127, *Certificado de Apresentação de Apreciação Ética*: 61039816.0.0000.5071 which is registered with the Brazilian Clinical Trials Registry under number U1111-1214-0568. All participants signed the informed consent form.

## Results

In total, 895 women were referred to undergo POP and LUTS by their attending physicians from January 2017 to December 2019. Of these women, 70 did not accept to participate in the study, 50 showed signs and/or symptoms of urinary UTI on the scheduled day of the urodynamic testing and 30 for not agreeing to sign the Free and Informed Consent Form. Therefore, 745 women participated in the present study. The chart 1 demonstrates Voiding Urodynamics Parameters, their Units, and Reference Values.

The chart 2 shows that the age of the patients ranged from 31 to 94 years, (mean of 61.7 years), with the predominant age range being between 50 and 69 years. The anterior compartment was the one with the greatest prolapse in the studied sample, with only 47% of women having Ba<0.

**Chart 2:** Distribution of patients according to age group, presence of previous pelvic surgeries, and POP-Q stage

Variables		n(%)
Age (years ± SD)		61.7 ± 11.8
Age group (n, %)		
	30 to 49 years	142(19.0)
	50 to 69 years	385(51.7)
	70 years or more	218(29.3)
Vaginal delivery (n ± SD)		2.1 ± 2.2
Cesarean delivery (n ± SD)		0.8 ± 1.1
Pelvic surgeries (n ± SD)		0.7 ± 0.9
Pelvic surgeries		
	No surgeries	469(63.6)
	Hysterectomy	124(16.6)
	Perineoplasty	136(18.2)
	Midurethral Sling	16(1.6)
POP-Q Ba		
	< 0	350(47.0)
	0	176(23.6)
	> 0	219(29.4)
POP-Q Bp		
	< 0	651(87.4)
	0	82(11.0)
	> 0	16(1.6)
POP-Q C		
	< 0	678(91.0)
	≥ 0	67(9.0)
POP-Q D		
	< 0	717(96.2)
	≥ 0	28(3.8)
POP		
	No symptomatic POP (without exteriorization)	510(68.4)
	Some POP (with exteriorization)	235(31.6)

n=745; n: number of individuals; SD: standard deviation; POP-Q: *Pelvic Organ Prolapse Quantification*

The chart 3 contains a description of the urodynamic data of the studied population.

**Chart 3:** Description of voiding urodynamics parameters

Parameters		Reference value	Mean	SD	Median
Qmax in uroflowmetry (mL/s)		> 12 mL/s	16.7	8.7	16
Post-void residual volume (mL)		< 100 mL or < 20% of maximum cystometric capacity	50.2	38.3	40
Maximum cystometric capacity (mL)		350 – 500 mL	363.7	107.2	360
Bladder compliance (mL)		≥ 30 mL/cmH_2_O	44.1	25.9	42
PdetQmax (cmH_2_O)		< 20 cmH_2_O	32.6	15.1	29
Qmax in the flow/pressure study (mL/s)		> 12 mL/s	15.0	7.7	14
**Variables**		**Reference value**			**n(%)**
Post-void residual volume classification					
	Normal	< 100 mL or < 20% of maximum cystometric capacity			476(63.9)
	Increased	> 100 mL or > 20% of maximum cystometric capacity			269(36.1)
Detrusor overactivity					
	Present	>16 cmH_2_O			256(34.4)
	Not present	< 16 cmH_2_O			461(61.9)
	Present at the end of micturition	>16 cmH_2_O			28(3.8)
Leakage due to detrusor overactivity					
	Absent	< 1 mL			579(77.7)
	Present	> 1 mL			166(22.3)
Urethral resistance					
	Normal	< 0,2 cmH_2_O/(mL/s)^2^			442(59.3)
	Increased	> 0,2 cmH_2_O/(mL/s)^2^			303(40.7)
Bladder outlet obstruction index					
	Non-obstructive	< 20			586(78.7)
	Intermediate	20 – 40			117(15.7)
	Obstructive	> 40			42(5.6)
Bladder contractility index (BCI)					
	Strong	> 150			97(13.0)
	Normal	100 – 150			315(42.3)
	Weak	< 100			333(44.7)
Blaivas & Groutz nomogram for female bladder outlet obstruction					
	No obstruction	Uroflowmetry > 20 mL/s			350(47.0)
	Mild obstruction	Uroflowmetry 10 – 20 mL/s			326(43.8)
	Moderate/severe obstruction	Uroflowmetry < 20 mL/s			69(9.2)

n=745; n: number of individuals; SD: standard deviation; Qmax in uroflowmetry: Maximum flow rate in uroflowmetry; PdetQmax: Detrusor pressure at maximum flow; Qmax in the flow/pressure study: Maximum flow in the flow/pressure study

Next, the POP-Q measurements were correlated with the voiding urodynamics parameters, the results of which are presented in charts 3, 4 and 5. For the "Ba>0" group, a higher mean age, vaginal births, post-void residue volume and PdetQmax were observed. The "Ba< 0" group had a higher average number of pelvic surgeries, Qmax in uroflowmetry, maximum cystometric capacity, bladder compliance and Qmax in the flow/pressure study. This behavior was similar for the Bp point groups, except for the parameters of maximum cystometric capacity and bladder compliance, which did not show statistical differences. Patients in the "C≥0" group had higher average ages, number of vaginal deliveries, post-void residue, bladder compliance and PdetQmax. Patients with "C<0" had higher averages for the number of cesarean deliveries, Qmax in uroflowmetry, maximum cystometric capacity and Qmax in the flow/pressure study. This finding was repeated for the "D>0" and "D<0" groups, with the except for the variables "cesarean section" and "maximum cystometric capacity", as they did not present statistical differences, and for "bladder compliance", whose average was higher in the "D<0" group (Chart 4).

**Chart 4:** Comparisons between points Ba, Bp, C, and D with patient characteristics

Variables	Ba	n	Mean ± SD	p-value*	Bp	n	Mean ± SD	p-value*	C	n	Mean ± SD	p-value**	D	n	Mean ± SD	p-value**
Age (years)	< 0	350	57.8 ± 10.4	<0.001	< 0	596	59.8 ± 11.2	<0.001	< 0	678	60.8 ± 11.4	<0.001	< 0	717	61.4 ± 11.7	0.001
	0	176	62.3 ± 11.2		0	124	69.1 ± 11.3		≥ 0	67	70 ± 12.9		≥ 0	28	69.4 ± 11.6	
	> 0	219	67.3 ± 12.04		> 0	25	69.2 ± 11.6									
Vaginal delivery (n)	< 0	350	1.5 ± 1.7	<0.001	< 0	596	1.7 ± 1.9	<0.001	< 0	678	1.9 ± 2.1	<0.001	< 0	717	2.1 ± 2.2	0.011
	0	176	2.3 ± 2.2		0	124	3.6 ± 2.9		≥ 0	67	3.8 ± 2.8		≥ 0	28	3.0 ± 2.2	
	> 0	219	3 ± 2.6		> 0	25	3.8 ± 2.1									
Cesarean delivery (n)	< 0	350	0.8 ± 0.9	<0.001	< 0	596	0.9 ± 1	0.008	< 0	678	0.8 ± 1	0.002	< 0	717	0.8 ± 1.1	0.114
	0	176	0.9 ± 1.0		0	124	0.7 ± 1.4		≥ 0	67	0.6 ± 1.4		≥ 0	28	0.5 ± 0.8	
	> 0	219	0.7 ± 1.3		> 0	25	0.6 ± 0.9									
Pelvic surgeries (n)	< 0	350	0.6 ± 0.9	<0.001	< 0	596	0.7 ± 0.9	0.004	< 0	678	0.7 ± 0.9	0.131*	< 0	717	0.7 ± 0.9	0.108
	0	176	0.8 ± 0.9		0	124	0.9 ± 0.9		≥ 0	67	0.8 ± 0.9		≥ 0	28	0.9 ± 0.7	
	> 0	219	0,8 ± 0,9		> 0	25	0.4 ± 0.7									
Qmax in uroflowmetry (mL/s)	< 0	350	21.3 ± 7.5	<0.001	< 0	596	18 ± 8.7	<0.001	< 0	678	17.5 ± 8.6	<0.001	< 0	717	16.9 ± 8.7	<0.001
	0	176	17.8 ± 7.8		0	124	11.7 ± 6.7		≥ 0	67	8.1 ± 3.7		≥ 0	28	11.6 ± 7.9	
	> 0	219	8.3 ± 3.6		> 0	25	8.8 ± 4.7									
Post-void residual volume (mL)	< 0	350	34.3 ± 15.6	<0.001	< 0	596	47.5 ± 37.1	<0.001	< 0	678	45.9 ± 32.8	<0.001	< 0	717	48.5 ± 34.9	<0.001
	0	176	38 ± 19.9		0	124	56.8 ± 41.9		≥ 0	67	93.5 ± 58.4		≥ 0	28	93.1 ± 79.2	
	> 0	219	85.5 ± 50.3		> 0	25	81.6 ± 31.1									
Maximum cystometric capacity (mL)	< 0	350	388 ± 99.8	<0.001	< 0	596	364.5 ± 108.5	0.385	< 0	678	365 ± 106.4	0.008	< 0	717	364.9 ± 106.4	0.189
	0	176	369 ± 101.3		0	124	356.2 ± 102.3		≥ 0	67	330.5 ± 110.5		≥ 0	28	333.6 ± 124.7	
	> 0	219	320.5 ± 110.6		> 0	25	382.8 ± 101.4									
Vesical compliance (mL/cmH_2_O)	< 0	350	47.2 ± 23.6	<0.001	< 0	596	43.7 ± 22	0.086	< 0	678	43.8 ± 22.9	0.030	< 0	717	44.3 ± 25.7	0.025
	0	176	43.9 ± 24.5		0	124	45.3 ± 36.7		≥ 0	67	47,4 ± 46,8		≥ 0	28	39.2 ± 33	
	> 0	219	39.4 ± 29.9		> 0	25	49.4 ± 44									
PdetQmax (cmH2O)	< 0	350	27.8 ± 1	<0.001	< 0	596	31.1 ± 14	<0.001	< 0	678	31.5 ± 14.6	<0.001	< 0	717	32,2 ± 15	<0.001
	0	176	30.4 ± 11.3		0	124	38.7 ± 18.8		≥ 0	67	43.4 ± 15,8		≥ 0	28	41.5 ± 15.1	
	> 0	219	42 ± 19.6		> 0	25	36.9 ± 10.2									
Qmax in the flow/pressure study (mL/s)	< 0	350	18.8 ± 6.8	<0.001	< 0	596	15.9 ± 7.9	<0.001	< 0	678	15.7 ± 7.7	<0.001	< 0	717	15.1 ± 7.7	0.002
	0	176	15.7 ± 7.6		0	124	11.5 ± 6.2		≥ 0	67	8 ± 3.5		≥ 0	28	11.5 ± 8	
	> 0	219	8.3 ± 3.6		> 0	25	9.4 ± 3.9									

n=745;

* Kruskal-Wallis test.

** Mann-Whitney U test.

Legend: n: number of individuals; SD: standard deviation; Qmax in uroflowmetry: Maximum flow rate in uroflowmetry; PdetQmax: Detrusor pressure at maximum flow; Qmax in the flow/pressure study: Maximum flow in the flow/pressure study

**Chart 5:** Comparisons between points Ba, Bp, C, and D with voiding urodynamics parameters

Variables	POP-Q Ba				POP-Q Bp				POP-Q C			POP-Q D		
	< 0	0	> 0	p-value	< 0	0	> 0	p-value	< 0	≥ 0	p-value	< 0	≥ 0	p-value
	n(%)	n(%)	n(%)		n(%)	n(%)	n(%)		n(%)	n(%)		n(%)	n(%)	
Post-void residual volume classification				<0.001*				<0.001*			<0.001*			<0.001*
Normal	319 (91.1)	137 (77.8)	20 (9.1)		425 (71.3)	50 (40.3)	1 (4.0)		471 (69.5)	5(7.5)		467 (65.1)	9 (32.1)	
Increased	31 (9.0)	39 (22.2)	199 (90.9)		171 (28.7)	74 (59.7)	24 (96.0)		207 (30.5)	62 (92.5)		250 (34.9)	19 (67.9)	
Qmax in uroflowmetry				0.005*				0.005*			0.005*			0.005*
≤ 12 cmH_2_O	34 (4.6)	49 (6.6)	192 (25.8)		76 (10.2)	74 (9.9)	125 (16.8)		213 (28.6)	62 (8.3)		214 (28.7)	8 (1.1)	
> 12 cmH_2_O	711 (95.4)	696 (93.4)	553 (74.2)		669 (89.8)	671 (91.1)	620 (83.2)		532 (71.4)	783 (92.7)		531 (71.3)	737 (98.9)	
Vesical compliance				0.005*				0.005*			0.005*			0.005*
< 30 cmH_2_O/mL	45 (6.1)	31 (4.2)	70 (9.4)		110 (14.8)	28 (3.8)	7 (0.94)		130 (17.5)	16 (2.2)		134 (17.9)	6 (0.8)	
≥ 30 cmH_2_O/mL	700 (93.9)	714 (95.8)	675 (90.6)		635 (85.2)	717 (96.2)	738 (99.0)		615 (82.5)	729 (97.8)		612 (82.1)	739 (99.2)	
Detrusor overactivity				0.017*				0.761*			0.700*			0.356*
Present	118 (33.7)	66 (37.5)	100 (45.7)		228 (38.3)	45 (36.3)	11 (44.0)		257 (37.9)	27 (40.3)		271 (37.8)	13 (46.4)	
Absent	232 (66.3)	110 (62.5)	119 (54.3)		368 (61.7)	79 (63.7)	14 (56.0)		421 (62.1)	40 (59.7)		446 (62.2)	15 (53.6)	
Leakage due to detrusor overactivity				<0.001*				0.003*			0.345*			0.082*
Absent	292 (83.4)	137 (77.8)	150 (68.5)		476 (79.9)	82 (66.1)	21 (84.0)		530 (78.2)	49 (73.1)		561 (78.2)	18 (64.3)	
Present	58 (16.6)	39 (22.2)	69 (31.5)		120 (20.1)	42 (33.9)	4 (16.0)		148 (21.8)	18 (26.9)		156 (21.8)	10 (35.7)	
Urethral resistance				<0.001*				<0.001*			<0.001*			<0.001*
Normal	296 (84.6)	123 (69.9)	23 (10.5)		396 (66.4)	42 (33.9)	4 (16.0)		437 (64.5)	5(7.5)		435 (60.7)	7 (25.0)	
Increased	54 (15.4)	53 (30.1)	196 (89.5)		200 (33.6)	82 (66.1)	21 (84.0)		241 (35.5)	62 (92.5)		282 (39.3)	21 (75.0)	
Bladder outlet obstruction index (BOOI)				<0.001*				<0.001**			<0.001**			0.003**
Non-obstructive	334 (95.4)	160 (90.9)	92 (42.0)		495 (83.1)	75 (60.5)	16 (64.0)		560 (82.6)	26 (38.8)		571 (79.6)	15 (53.6)	
Intermediate	14 (4.0)	14 (8.0)	89 (40.6)		71 (11.9)	39 (31.5)	7 (28.0)		88 (13.0)	29 (43.3)		110 (15.3)	7 (25.0)	
Obstructive	2 (0.6)	2 (1.1)	38 (17.4)		30 (5.0)	10 (8.1)	2 (8.0)		30 (4.4)	12 (17.9)		36 (5.0)	6 (21.4)	
Bladder contractility index (BCI)				<0.001*				<0.001**			<0.001*			0.214**
Strong	71 (20.3)	19 (10.8)	7 (3.2)		90 (15.1)	7 (5.6)	0 (0.0)		96 (14.2)	1 (1.5)		94 (13.1)	3 (10.7)	
Normal	187 (53.4)	77 (43.8)	51 (23.3)		263 (44.1)	46 (37.1)	6 (24.0)		298 (44.0)	17 (25.4)		307 (42.8)	8 (28.6)	
Weak	92 (26.3)	80 (45.5)	161 (73.5)		243 (40.8)	71 (57.3)	19 (76.0)		284 (41.9)	49 (73.1)		316 (44.1)	17 (60.7)	
Blaivas-Groutz nomogram for female bladder outlet obstruction				<0.001*				<0.001**			<0.001*			0.014**
No obstruction	252 (72.0)	94 (53.4)	4 (1.8)		320 (53.7)	28 (22.6)	2 (8.0)		349 (51.5)	1 (1.5)		344 (48.0)	6 (21.4)	
Mild obstruction	96 (27.4)	74 (42.0)	156 (71.2)		238 (39.9)	69 (55.6)	19 (76.0)		274 (40.4)	52 (77.6)		309 (43.1)	17 (60.7)	
Moderate/severe obstruction	2 (0.6)	8 (4.5)	59 (26.9)	<0.001*	38 (6.4)	27 (21.8)	4 (16.0)		55 (8.1)	14 (20.9)		64 (8.9)	5 (17.9)	

n=745;

* Chi-square test.

** Likelihood Ratio.

Legend: n: number of individuals; POP-Q: Pelvic Organ Prolapse Quantification; Qmax in uroflowmetry: Maximum flow rate in uroflowmetry

Voiding urodynamics parameters suggestive of BOO diagnosis found in women with POP were: maximum flow rate (Qmax) in the uroflowmetry ≤12mL/s; detrusor pressure at maximum flow (PdetQmax) >20cmH2O; and increased urethral resistance, bladder outlet obstruction index (BOOI) intermediate and obstructive, weak bladder contractility index (BCI) and Blaivas & Groutz nomogram with obstruction (Chart 5).

When evaluating the differences between patients with and without POP, it was observed that those who presented some type of POP were mean age (y) older (67.6 X 58.9); had higher post-void residue volume (mL) (85.9 X 33.9); higher PdetQmax (cmH_2_O) values (41.3 x 28.5); lower Qmax (mL/s) values on uroflowmetry (8.5 X 20.4), lower maximum cystometric capacity (mL) (325.7 X 381.2), lower bladder compliance (mL/cmH2O) (39.8 x 46.1) and lower Qmax (mL/cmH2O) in the flow/pressure study (8.4 X 18.0) (*p*<0.001) (Chart 6).

**Chart 6:** Voiding urodynamics parameters based on the presence or absence of Pelvic Organ Prolapse (POP) exteriorization

Variables		Without POP		With POP		p-value
Age (years ± SD)		58.9	10.7	67.6	12.0	< 0.001*
Qmax in uroflowmetry (mL/s ± SD)		20.4	7.7	8.5	3.8	< 0.001*
Post-void residual volume (mL ± SD)		33.9	14.4	85.6	48.9	< 0.001*
Maximum cystometric capacity (mL ± SD)		381.2	100.7	325.7	111.3	< 0.001*
Bladder compliance (mL/cmH_2_O ± SD)		46.1	23.1	39.8	30.9	< 0.001*
PdetQmax (cmH_2_O ± SD)		28.5	10.5	41.3	19.2	< 0.001*
Qmax in the flow/pressure study (mL/s ± SD)		18.0	7.2	8.4	3.7	< 0.001*
**Variables**		**n(%)**		**n(%)**		**p-value**
Post-void residual volume						
	Normal	455(89.2)		21(8.9)		< 0.001**
	Increased	55(10.8)		214(91.1)		
Detrusor overactivity						
	Present	157(30.8)		99(42.1)		0.003
	Absent	353(69.2)		136(57.9)		
Leakage due to detrusor overactivity						
	Present	415(81.4)		164(69.8)		< 0.001**
	Absent	95(18.6)		71(30.2)		
Urethral resistance						
	Normal	414(81.2)		28(11.9)		< 0.001**
	Obstructive	96(18.8)		207(88.1)		
Bladder outlet obstruction index (BOOI)						
	Non-obstructive	481(94.3)		105(44.7)		< 0.001**
	Intermediate	25(4.9)		92(39.1)		
	Obstructive	4(0.8)		38(16.2)		
Bladder contractility index (BCI)						
	Strong	90(17.6)		7(3.0)		< 0.001**
	Normal	262(51.4)		53(22.6)		
	Weak	158(31.0)		175(74.5)		
Blaivas & Groutz nomogram for female bladder outlet obstruction						< 0.001**
	No obstrucion	344(67.5)		6(2.6)		
	Mild obstruction	156(30.6)		170(72.3)		
	Moderate/severe obstruction	10(2.0)		59(25.1)		

n=745;

* Mann-Whitney U test.

** Chi-square test.

Legend: n: number of individuals; SD: standard deviation; Qmax in uroflowmetry: Maximum flow rate in uroflowmetry; PdetQmax: Detrusor pressure at maximum flow; Qmax in the flow/pressure study: Maximum flow in the flow/pressure study

## Discussion

The scope of this study was to characterize the urodynamic parameters of voiding dysfunction in women with and without POP, with the aim of offering variables that suggest the diagnosis of BOO.^(18,19)^ To this end, a group of patients with a ‘Ba > 0’ point was observed, indicating a pattern of BOO through voiding urodynamics parameters that might suggest detrusor hypoactivity. The suspicion of detrusor hypoactivity should be considered in POP cases.^(19-22)^

In the present study, urodynamic data obtained without reduction of POP were analyzed, even though Sierra (2019)^(20)^ noted that reducing POP during urodynamics to investigate urinary incontinence could avoid unnecessary concomitant procedures. However, this approach does not allow for the assessment of BOO.^(23)^

Importantly, the criteria for classifying BOO used in the present investigation followed the male parameters and the female nomogram of Blaivas et al.^(14)^ These assessments, compared with those of the POP-Q groups at points Ba, Bp, C, and D, showed associations with various parameters in the urodynamic study, as reported by other authors.^(14,18,24)^ Notably, in the ‘> 0’ group, there was a greater frequency of Qmax in uroflowmetry < 12 mL/s, PdetQmax in the flow/pressure study > 20 cmH2O, increased urethral resistance, elevated postvoid residual volume, intermediate and obstructive results for the BOOI, a weak bladder contractility index, and a Blaivas & Groutz female obstruction nomogram indicating mild, moderate, and severe obstruction.^(24)^

Such findings demonstrated an important objectivity for the diagnosis of obstruction.^(25)^ Nitti (2005)^(12)^ considers that a Qmax in uroflowmetry with values below 10 mL/s and increased post-void residual volume are simple tests for the diagnosis of BOO, but they are not definitive parameters. Rademakers et al. ^(26)^ considered a volume of 20% of the maximum cystometric capacity or 100 mL as clinically significant for increased post-void residual volume. Hoffman et al. ^(25)^ found that up to 58% of voiding dysfunction with obstructive patterns occurred in women with advanced POP (stages III and IV). The presence of detrusor overactivity is significant among patients who exhibited the criteria for BOO, and a considerable number of them experienced leakage associated with detrusor overactivity in the groups with points Ba, Bp, C, and D ‘> 0’.^(15)^

Scientific literature suggests that the results found in the BOOI differ between men and women due to the higher observed PdetQmax values in the male flow/pressure study compared to the female flow/pressure study. These findings indicate that female urethral resistance has lower values than male urethral resistance.^(24,27)^ In this context, the findings of the present study support this information. Most patients showed PdetQmax in the flow/pressure study <20 cmH2O in the ‘<0’ group at points C, D, Ba, and Bp. This value aligns with the standard considered one of the criteria for the absence of BOO. In the same analysis, PdetQmax values in the flow/pressure study ≤10 cmH2O were added as a criterion for suspecting detrusor hypoactivity in the Ba’<0’, ‘0’, and ‘0’ groups,^(19)^ the only group with statistical numerical representation for this analysis, with this parameter observed in 17.9% of the ‘>0’ group.

Furthermore, when male parameters were used, it was observed that in the ‘> 0’ groups, decreased bladder compliance occurred more frequently. Losses due to detrusor overactivity were more frequent in the ‘>0’ groups, increased urethral resistance and ‘weak’ bladder contractility index were present in patients from the ‘≥0’ and ‘>0’ groups and normal at points C, D, Ba, and Bp ‘<0’. With these urodynamic values, the presence of BOO was demonstrated in groups C and D ‘≥0’ and the absence of BOO in groups C and D ‘<0’ and at points Ba and Bp ‘<0’ and ‘0’, by associating POP-Q with urodynamic variables, as discussed in the scientific literature.^(28)^

Rademakers et al.^(26)^ reported that between 14% and 22% of symptomatic patients with LUTS may have mild or moderate BOO. In the present investigation, we found 62 women who met all the defined obstruction criteria, and all were from the ‘>0’ and ‘≥ 0’ groups, with percentage values close to those reported by other authors.^(29)^ Additionally, 39 patients were found in the ‘Ba>0’ group with all the factors indicating bladder impairment, meaning they were diagnosed with BOO and exhibited decreased bladder compliance, the presence of detrusor overactivity and/or leakage from detrusor overactivity, low detrusor pressure values in the flow/pressure study, and increased post-void residual, corresponding to 17.9% of this group. These patients might progress to a case of detrusor underactivity.

The Qmax in uroflowmetry and the Qmax in the flow/pressure study were obstructive (average 10 mL/s), the PdetQmax in the flow/pressure study was increased, post-void residual volume was elevated in 66.1%, the Blaivas & Groutz female obstruction nomogram was mildly obstructive in 55.6%, and the bladder contractility index was weak in 57.3%. However, there was no detrusor overactivity-related leakage, and a non-obstructive BOOI was found in 60.5%. These findings in the ‘Bp=0’ group, which closely resemble the values observed in the ‘Bp>0’ group with an obstructive pattern in the studied variables, might be due to rectocele compression on the urethra. In this case, a new study with posterior prolapse reduction could clarify whether there is BOO in the ‘0’ group before planning therapeutic approaches for these patients. The parameters obtained in this research for point C showed that BOO was demonstrated in the ‘C≥0’ group, where the POP possibly causes dynamic alterations in urethral anatomy and functionality, leading to angulation and concurrent obstruction, which was not observed in the ‘C<0’ group.^(6,24)^

In the apical evaluation of the POP-Q classification, at point D, it was observed that the parameters in the ‘D≥0’ group clearly indicated the presence of BOO, similar to what was observed at point C, confirming the hypothesis that apical POP significantly affect the quality of micturition.^(7)^

In a second phase of analysis, after reclassifying the patients into a control group (absence of prolapse exteriorization) and a group with prolapse (presence of anterior vaginal wall prolapse exteriorization), the same obstructive patterns were found in the prolapse exteriorization group. In this group, there was a higher presence of increased post-void residual volume, detrusor overactivity and leakage due to detrusor overactivity, a greater number of intermediate and obstructive BOO indices, a more pronounced weak bladder contractility index, and an obstructive Blaivas & Groutz female obstruction nomogram.^(21)^

It was found that patients with points Ba and Bp ‘> 0’ and C/D ‘≥ 0’ are highly likely to have BOO and may also have detrusor overactivity more frequently than patients with points Ba, Bp, C, and D ‘<0’.^(30)^ Furthermore, additional studies with prolapse reduction could be conducted to define more specific criteria and nomograms for BOO in apical compartments and in Bp of POP-Q, as well as for the differential diagnosis of detrusor overactivity in Ba, Bp, and apical points.^(31)^

Finally, the findings reaffirm that the voiding urodynamics parameters listed in this study were significant in cases of isolated anterior vaginal wall prolapse ‘Ba>0’ or associated with apical prolapses ‘C’ and D ≥0’.

The findings of the present study allow us to conclude that lower Qmax in uroflowmetry, maximum cystometric capacity, bladder compliance, Qmax in the flow/pressure study, increased post-void residual volume and PdetQmax, elevated urethral resistance, increased Qmax in uroflowmetry and bladder compliance, absence of detrusor overactivity, BOOI, and the Blaivas & Groutz female obstruction nomogram with some degree of obstruction, along with a weak bladder contractility index, were suggestive of BOO in POP.

The aim of this study was to determine urodynamic parameters to facilitate the diagnosis of BOO in patients with symptomatic pelvic organ prolapse without reducing prolapse. Data on these parameters are scarce because when urodynamic studies are performed, most publications report data on BOO in patients with reduced prolapse, which mimics asymptomatic patients.

The main limitation was the lack of robust data on the topic in the literature to encourage discussion. Another limitation refers to heterogeneity of the groups and the absence of a urodynamic evaluation with and without POP reduction as an internal comparison parameter.

## Conclusion

The voiding urodynamics parameters listed in this study allows the evaluation of variables suggestive of BOO diagnosis in women with and without POP. Future studies with and without prolapse reduction and/or videourodynamics may confirm or refute the suspicion of bladder outlet obstruction according to the findings described.
